# Microarray Analysis of the Intestinal Host Response in *Giardia duodenalis* Assemblage E Infected Calves

**DOI:** 10.1371/journal.pone.0040985

**Published:** 2012-07-27

**Authors:** Leentje Dreesen, Manuela Rinaldi, Koen Chiers, Robert Li, Thomas Geurden, Wim Van den Broeck, Bruno Goddeeris, Jozef Vercruysse, Edwin Claerebout, Peter Geldhof

**Affiliations:** 1 Department of Virology, Parasitology and Immunology, Faculty of Veterinary Medicine, Ghent University, Merelbeke, Belgium; 2 Department of Pathology, Bacteriology and Avian Diseases, Faculty of Veterinary Medicine, Ghent University, Merelbeke, Belgium; 3 Bovine Functional Genomics Laboratory, Animal and Natural Resources Institute, USDA-ARS, Beltsville, Maryland, United States of America; 4 Department of Morphology, Faculty of Veterinary Medicine, Ghent University, Merelbeke, Belgium; Centro de Investigacion y de Estudios Avanzados del Instituto Politecnico Nacional, Mexico

## Abstract

Despite *Giardia duodenalis* being one of the most commonly found intestinal pathogens in humans and animals, little is known about the host-parasite interactions in its natural hosts. Therefore, the objective of this study was to investigate the intestinal response in calves following a *G. duodenalis* infection, using a bovine high-density oligo microarray to analyze global gene expression in the small intestine. The resulting microarray data suggested a decrease in inflammation, immune response, and immune cell migration in infected animals. These findings were examined in more detail by histological analyses combined with quantitative real-time PCR on a panel of cytokines. The transcription levels of IL-6, IL-8, IL-13, IL-17, and IFN-γ showed a trend of being downregulated in the jejunum of infected animals compared to the negative controls,.No immune cell recruitment could be seen after infection, and no intestinal pathologies, such as villus shortening or increased levels of apoptosis. Possible regulators of this intestinal response are the nuclear peroxisome proliferator-activated receptors alpha (PPARα), and gamma (PPARγ) and the enzyme adenosine deaminase (ADA), all for which an upregulated expression was found in the microarray and qRT-PCR analyses.

## Introduction

The protozoan parasite *Giardia duodenalis* (also known as *G. intestinalis* and *G. lamblia*) is one of the most commonly found intestinal pathogens in a wide range of vertebrate hosts. In humans, an estimated 280 million infections occur yearly, especially in developing countries. In addition, *G. duodenalis* is a common parasite in both farm and companion animals [1]. Several studies have shown that for cattle, sheep, and goats farm prevalences can be as high as 100% [2].

An infection with *G. duodenalis* can present itself with gastro-intestinal complaints such as diarrhea, although infections often remain asymptomatic and therefore unnoticed by the carrier. Younger individuals are especially at risk of developing clinical giardiasis, and chronic infections can lead to malnutrition, growth impairment, and, in the case of humans, poor cognitive development [3], [4], [5]. Both acute and chronic infections, whether symptomatic or not, have been described in earlier studies. In man, experimental infection with cysts led to spontaneous disappearance of cyst excretion in faeces after a period ranging from 5 to 41 days. In 2 persistent cases, cyst excretion lasted at least 129 days [6]. In longitudinal studies on children in Israel [7], Brazil [8] and Peru [9], infections were chronic with prolonged cyst excretion. In addition, infections were recurrent with reappearance of the parasite after treatment. In cattle, the general consensus is that infections are chronic and reoccuring as shown by cyst excretions lasting well over 100 days in both dairy and beef cattle [2]. Chronic infections are also documented in dogs [10], goats [11], and sheep [12].

Our knowledge of the immune response against *G. duodenalis* is largely based on *in vitro* studies and infection trials in mice with a human-derived axenised *G. duodenalis* assemblage B isolate or *G. muris*. Elements of the innate immune response, such as the production of nitric oxide, mucins, defensins, and other antimicrobial peptides, were shown to have a negative effect on trophozoites *in vitro* [13]. In the presence of live parasites and parasite extracts *in vitro*, murine bone marrow derived dendritic cells were inhibited in eliciting an LPS-induced Th1 inflammatory pathway due to decreased IL-12 and increased IL-10 production [14]. Further downstream, T-cell dependent mechanisms seem essential in the control of murine giardiasis, as shown by the development of chronic infections with *G. duodenalis* in nude mice, mice treated with anti-CD4, and mice lacking the T cell receptor β gene [15]. In terms of cytokines, IL-6 was observed to be produced by DC’s in the presence of live parasite and parasite extract [16]. Production of TNF-α, IFN-γ, IL-4, IL-10, IL-13, IL-17 and IL-22 by mouse lymph node cells in response to parasite extract [17] is also described, while in humans elevated levels of IL-5, IL-6 and IFN-γ were seen in the sera of infected adults [13]. The recruitment and activation of mucosal mast cells also appears to play an important role, as shown by the development of chronic giardiasis in *G. duodenalis* infected mast cell deficient mice. These mast cells can help B cell class switching to parasite specific IgA and contribute to changes in intestinal motility by influencing smooth muscle contractions following infection [13]. Another proposed effector mechanism is trophozoite phagocytosis by macrophages, since human monocytes and macrophages ingested and killed *G. duodenalis* trophozoites *in vitro* [18].

The extent to which the results obtained from the *in vitro* experiments or mice infection trials can be extrapolated to the *in vivo* situation in natural hosts is still unclear. Our knowledge of *in vivo* immune response in natural hosts is limited to a number of studies in which systemic antibody and cytokine responses were investigated as well as the long-term cellular immune response in humans [19]. So despite the global importance of this parasite, there is little information available on the intestinal host-parasite interactions in natural hosts that can explain the often chronic and recumbent character of giardiasis. Therefore, the objective of this study was to investigate the intestinal response in calves following a primary *G. duodenalis* infection by histological and gene expression analysis.

## Materials and Methods

### Infection Trial and Tissue Collection

This study was conducted in accordance with the E.U. Animal Welfare Directives and VICH Guidelines for Good Clinical Practice, and ethical approval to conduct this study was obtained from the Ethical Committee of the Faculty of Veterinary Medicine, Ghent University.

Eight male Holstein calves aged two to four weeks were used for the trial. Prior to arrival, all animals were screened three consecutive days for the presence of *Giardia* cysts and *Cryptosporidium* oocysts in their faecal samples with the use of the commercially available MERIFLUOR *Cryptosporidium/Giardia* immune-fluorescence assay (IFA) (Meridian diagnostics Inc., Cincinnati, OH). In addition, the animals were checked for the presence of Bovine Viral Diarrhea antigen in their blood, while faecal samples were screened for the presence of *Eimeria spp*. oocysts (McMaster method). After confirming their negative status for all these pathogens, four randomly chosen animals were placed in a pen in which *Giardia*-excreting calves had been housed prior to this experiment. The four remaining animals were kept as negative controls in separate *G. duodenalis*-free stables. All calves in the study received the same commercial milk replacer (Spraystart-Z® from Aveve; 6L per calf/day). Water and hay were provided *ad libitum* throughout the experiment. After three weeks, the presence or absence of a *G. duodenalis* infection was confirmed by IFA in faecal samples from all calves after which the animals were euthanized.

Jejunal tissue samples for gene expression analysis and histology were taken 3 meters from the pylorus. For RNA extraction, tissue was immediately snap frozen in liquid nitrogen and stored at -80°C. The same procedure was followed for the draining mesenterial lymph nodes. For histology, tissue was fixed in 10% formaldehyde in phosphate buffered saline (PBS) for 24 h, followed by incubation for 1 h in distilled water and storage in 70% ethanol, all at room temperature. The samples were subsequently dehydrated through a series of graded ethanol solutions followed by xylene and embedded in paraffin.

### Molecular Characterization

Faecal samples were collected in order to genotype *G. duodenalis* present in the infected animals. DNA was extracted using the QIAamp Stool Mini Kit (Qiagen) according to the manufacturer’s instructions, incorporating an initial step of three freeze–thaw cycles (freezing in liquid nitrogen for 5 min and heating at 95°C for 5 min) into the protocol to maximize cyst lysing. The eluted DNA was dissolved in 15 µl of ultra-pure water.

A nested PCR targeting the triosephosphate isomerase (*tpi*) gene was used for specific amplification of assemblage A and E, as described by Geurden et al. [20].

### RNA Extraction

Extraction of total RNA from tissue samples was done using TRIzol (Invitrogen) followed by further purification with the RNeasy Mini kit (Qiagen) following the manufacturer’s instructions. An on-column DNase digestion was performed using the RNase-free DNase set (Qiagen) to remove any contaminating genomic DNA. Total RNA concentrations were determined using a NanoDrop ND-1000 spectrophotometer (NanoDrop Technologies) and the quality of the RNA was verified with an Experion™ Automated Electrophoresis System (Bio-Rad). For all samples, the RNA quality indicator (RQI) calculated by the Experion™ software (Bio-Rad) was >8.0, indicating high RNA integrity.

### Microarray Analysis and Ingenuity Pathway Analysis

The RNA extracted from the jejunum of all the animals was used for microarray analysis. Double-stranded cDNA was synthesized using the Superscript™ Double-Stranded cDNA Synthesis kit (Invitrogen) followed by further concentration using the DNA Clean and Concentrator™-25 (Zymo Research) following manufacturer’s instructions. Microarray (Roche Nimblegen, Inc., Madison, WI) design and hybridization procedures were followed as previously described [21]. The bovine microarray consisted of 86,191 unique 60 mer oligonucleotides, which represents 45,383 bovine sequences or genes. Each unique oligonucleotide was repeated four times on the microarray. After hybridization, scanning, and image acquisition, data extraction from the raw images was executed using NimbleScan software (NimbleGen, Madison, WI). For each feature, relative signal intensities (log2) were generated using the robust multi-array average algorithm [22]. Further processing of the data was done based on the quantile normalization method [23]. The background-adjusted, normalized and log-transformed intensity values were further analyzed using MeV v4.2 (http://www.tm4.org/mev/). Raw and processed microarray data were deposited to the NCBI Gene Expression Omnibus (GEO) database (Accession# GSE35920).

The Ingenuity Pathway Analysis (IPA) software V6.0 (Ingenuity® Systems, www.ingenuity.com) was used to organize the genes regulated during the infection into networks of interacting molecules. The gene identifiers of the genes with a statistically significant change in expression (p<0,01 and p<0,05) and with a calculated positive or negative fold change of at least two-folds were uploaded in the software. These genes, called focus genes, were overlaid onto a global molecular network developed from information contained in Ingenuity’s Knowledge Base. Networks were then algorithmically generated based on their connectivity. Each network is assigned a score, a numerical value that ranks the networks according to how relevant they are to the genes in the uploaded dataset based on the number of focus genes and the size of the network. In addition, IPA was used for a functional analysis to identify the biological functions that were most significant to the uploaded datasets. Right tailed Fisher’s exact test was used to calculate a p-value determining the probability that each biological function assigned to that dataset is due to chance alone. The same method is used to analyze the biological functions of a calculated network.

### Quantitative Real-time PCR

A quantitative Real-time PCR (qRT-PCR) approach was used to validate the generated microarray data. The gene expression data of five upregulated and two downregulated genes were used as endogenous controls and analyzed with qRT-PCR. These genes include ATP-binding cassette sub-family G (WHITE) member 8 (ABCG8), adenosine deaminase (ADA), fatty acid synthase (FASN), peroxisome proliferator-activated receptor alpha (PPARα), peroxisome proliferator-activated receptor gamma (PPARγ), RAS guanyl releasing protein 2 (RASGRP2) and ras homolog gene family, member D (RHOD). In addition, qRT-PCR was also used to further elucidate the host-response during infection by analyzing expression of genes coding for the following cytokines: INF-γ, IL-1β, IL-4, IL-6, IL-8, IL-10, IL-13, IL-17, TGF-β1 and TNF-α.

The qRT-PCR analyses were performed using the SYBR Green Master Mix (Applied Biosystems) on cDNA samples produced using the iScript cDNA synthesis kit following the manufacturer’s instructions. Two µl of single stranded cDNA (10 ng of the input total RNA equivalent) and 500 nM of amplification primer were used in a reaction volume of 20 µl. The primer sets used to amplify the different bovine genes were designed using Primer3 software (http://frodo.wi.mit.edu/primer3/) and are listed in Table S1.

A StepOnePlus Real-Time PCR system (Applied Biosciences) executed the amplification cycles as follows: 95°C for 20 s; 35 cycles of 95°C for 5 s; and optimal annealing temperature (Ta) for 30 s. For all genes tested, the Ta was set at 60°C, with the exception of IL-4 where the Ta was 64°C. Reaction efficiencies were measured based on a standard dilution curve obtained by serial dilutions of pooled cDNA material from all the samples. To ensure specificity of the primers, melting curve analyses were performed at the end of the reactions, and the obtained PCR products were sequenced. In every assay, cDNA samples were analyzed in duplicate, and a non-template control was added. The obtained Ct values were transformed in relative quantities using the delta Ct method, which applies following formula: Q = E^(min Ct-sampleCt)^. In this formula, Q represents the relative quantity for each sample, E the amplification efficiency of the run, min Ct is the lowest Ct value among all the samples for each gene analyzed and sampleCt is the Ct for each sample in the run (http://medgen.ugent.be/~jvdesomp/genorm/geNorm_manual.pdf). These quantities were normalized against internal control genes, referred to as housekeeping genes (HKGs). The correct HKG were selected out of a panel of 6 candidate genes (beta actin (*ACTB)*, hypoxanthine phosphoribosyltransferase 1 (*HPRT1*), ribosomal protein P0 (*RPLP0*), succinate dehydrogenase flavoprotein subunit A (*SDHA*), TATA box binding protein (TBP)-associated factor (*TAF2*), and ubiquitin-conjugating enzyme E2D2 (*UBE2D2*) based on the gene stability measure M calculated by the GeNorm (geNorm3.5) software using the developers recommendations [24]. This identified UBE202 and ACTB as the most suitable HKG for jejunal samples and RPLP0 and HPRT1 for tissue of mesenterial lymph nodes.

Statistical analysis was carried out using GraphPad Prism software. The Nonparametic Whitney U test was used to determine differences between the infected and the control group. A P-value ≤0,05 was considered significant.

### Histology

Jejunal tissue sections of 4 µm were cut and mounted on APES-coated glass slides. Immunohistochemical stainings with a polyclonal rabbit anti-human CD3 (Dakocytomatio A/S, Glosturp, Denmark), a rabbit anti-human CD-20 (Neomarkers, CA, USA), and a monoclonal mouse anti-human MAC387 (Serotec, Cergy Saint-Christophe, France) were performed to identify and count T-cells, B-cells and macrophages, respectively, as previously described by Vangeel et al. (2011) [25]. Briefly, this included the use of the peroxidase streptavidin complex (Dakocytomatio A/S, Glosturp, Denmark), diaminobenzidin tetrahydrochloride (DAB, Sigma-Aldrich, St. Louis, USA), and H_2_O_2_ followed by counterstaining with haematoxylin. Quantification was done by counting positive cells in the lamina propria of a villus and associated crypt with light microscopy (Olympus BS 61, Olympus Belgium, Aartselaar, Belgium), via a 40× objective (400× magnification). Five appropriate areas were chosen randomly on each slide and cells were expressed as number of cells per 10^5^ µm^2^. Intraepithelial lymphocytes (IEL) were calculated by counting the number of CD3 positive lymphocytes per 100 enterocytes along the villus at 400× magnification. For this, at least 500 enterocytes were counted per sample.

To quantify the number of eosinophils and mast cells in the jejunal mucosa, tissue sections were stained with haematoxylin-eosin (H&E) and Giemsa, respectively. For each cell type, 10 non-overlapping high power fields (HPF) (400x) were checked and numbers were summed up to give a total cell count per 10 HPF.

To quantify apoptosis, apoptotic cells were counted using fluorescence microscopy (400x) on tissue slides (4 µm) stained with the DeadEnd™ Fluorometric TUNEL system (Promega). Staining was performed following the manufacturer’s recommendations. Counterstaining of the nuclei was done using DAPI (4′, 6-diamidino-2-phenylindole, dilactate; 1∶1000 in PBS; Invitrogen) for 5 minutes at room temperature. Five appropriate areas were chosen randomly on each slide, and apoptotic cells were expressed as number of cells per 10^5^ µm^2^.

Finally, the length of the villi and depth of the crypts in the jejunum were measured by analyzing 15 villi and their corresponding crypts under a microscope using a calibrated micrometer at 200× magnification. All results were compared by the student’s *t*-test using GraphPad Prism software. A P-value ≤0,05 was considered significant.

## Results

### Infection Trial

After 3 weeks of environmental exposure to *G. duodenalis* cysts, all four animals in the infected group tested positive for *Giardia* cysts in their faeces. Mean cyst excretion varied between 3950 and 15,000 cysts per gram (CPG) of faeces with an average of 10,328 CPG. Genotyping of the collected cysts revealed that animals were infected with the livestock-specific *G. duodenalis* assemblage E. The control group remained negative throughout the study.

### Transcriptomic Profiles and Pathway Analysis

A bovine high-density oligo microarray was used to analyze global gene expression in the small intestine of the calves. At a 95% confidence level (P<0,05), 140 genes were differentially expressed after infection with a minimum two-fold change (Table S2), 57 genes were transcriptionally downregulated, and 83 genes were upregulated.

**Figure 1:**
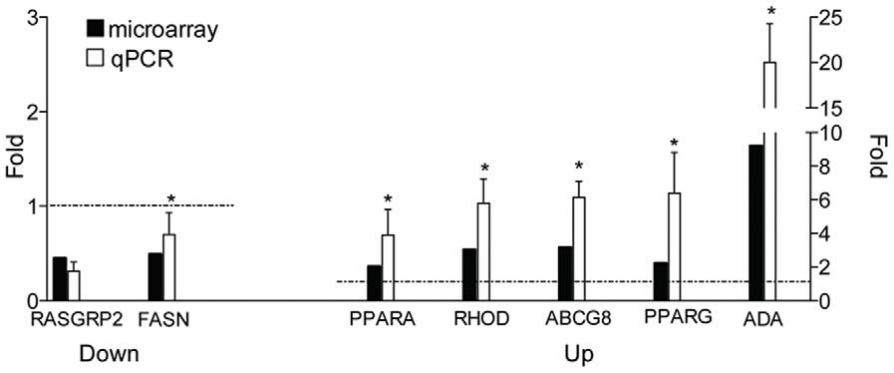
qRT-PCR validation of selected differentially expressed genes identified by microarray. Quantitative PCR on a panel of 7 genes was used to verify the transcriptional changes observed by microarray analysis. Consistent with the microarray results, expression of PPARA, RHOD, ABCG8, PPARG and ADA was upregulated when comparing infected animals to controls, while a downregulation could be seen for RASGRP2 (although not significant) and FASN. (* P<0.05).

The transcription levels of 7 selected genes were subsequently verified by quantitative PCR. Consistent with the microarray results, the expression of PPARα and PPARγ, ATP-binding cassette transporter (ABCG8), adenosine deaminase (ADA), and Ras homolog gene family member D (RHOD) was upregulated in the infected animals compared to controls, while a downregulation was confirmed for Ras guanyl releasing protein 2 (RASGRP2) and fatty acid synthase (FASN) (Figure 1); however, the downregulation of RASGRP2 as measured by qRT-PCR was not significant.

The IPA software was subsequently used to further examine the microarray dataset. When the dataset was uploaded, IPA determined the biological functions related to this dataset and gave the predicted activity of these functions. Overall, 4 main categories of functions were associated with the uploaded dataset: migration of leukocytes, inflammation, immune response, and lipid metabolism (summarized in Table 1). A total of 19 genes were related with cellular migration of B-cells, T-cells, phagocytes, and granulocytes, a function for which IPA predicted decreased activity. Two other functions impacted by the infection are part of the lipid metabolism. These include a decrease in lipid quantity (10 genes involved) as well as a decreased production of eicosanoids (5 genes involved). Finally, two functions labeled as ‘immune response’ and ‘inflammation’ both had a predicted decreased activity, with involvement of respectively 19 and 11 genes. The analysis also indicated that 4 genes were involved in all of the impacted functions, i.e. ADA, NOS2 (nitric oxide synthase 2), PPARα, and PPARγ (Table 1).

**Table 1:** Impacted biological functions and associated genes during *Giardia duodenalis* infection in calves as calculated by IPA.

Function	Prediction by IPA	Genes involved upregulated	Genes involved downregulated
Inflammatory response	Decreased	ADA, NT5E, PPARA, PPARG,	NOS2, IL12RB1, CXCL13,
		CCL14, PLA2G1B, FGG	DEFB4A
Immune response	Decreased	ADA, NT5E, PPARA, PPARG,	HSPH1, MADCAM1, IL12RB1,
		PLA2G1B, CCL14, FGG,	CXCL13, DEFB4A, CD79B,
		PLXNA1	ITGA4, CD1B, NOS2, MS4A1,
			POU2AF1
Migration of leukocytes	Decreased	ADA, NT5E, PPARA, PPARG,	MADCAM1, NOS2, IL12RB1,
		PLA2G1B, CCL14, ALB	CXCL13, RASGRP2, SH2D3C,
			F13A1, ITGA4, CXCR5,
			DEFB4A, POU2AF1, MAP4K1
Quantity of lipid	Decreased	AQP7, ADA, PPARA, HPGD,	NOS2
		PLA2G1B, ALB, PPARG,	
		LRAT, ABCG8	
Production of eicosanoid	Decreased	HPGD, PLA2G1B	NOS2, FASN, DEFB4

**Figure 2:**
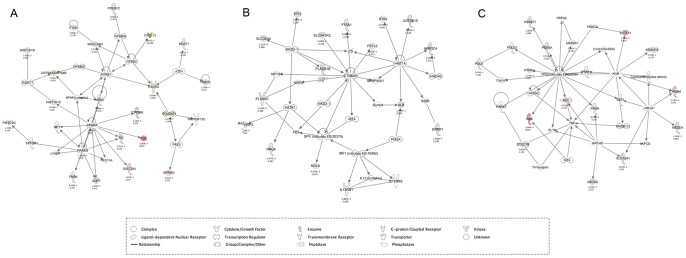
Regulatory networks impacted in the jejunum of *Giardia duodenalis* infected calves. (A) Score of 39. The network functions include immune cell trafficking and cell movement. (B) Score of 25. Functions include tissue morphology, cellular development, cellular growth and proliferation. (C) Score of 23. Functions include lipid metabolism, molecular transport and small molecule biochemistry. Color code: red, upregulated gene; green, downregulated gene. The degree of up- or downregulation is represented by the intensity of the colors, ranging from 2-fold to 20-fold change.

Three networks were calculated by IPA based on the uploaded p<0.05 dataset with a score higher then 20, implying that there is a probability of 10^−20^ that the genes in this network are solely associated by chance. The highest scoring network (score of 39) again deals with immune cell trafficking with a central role for the PPAR genes (Figure 2 panel A). Network 2 had a score of 25 and is associated with tissue morphology, cellular growth/proliferation, and cellular development. Finally, the network functions of the third network (score of 23) included lipid metabolism, molecular transport, and small molecule biochemistry.

### Analysis of the Intestinal Immune Response

**Figure 3:**
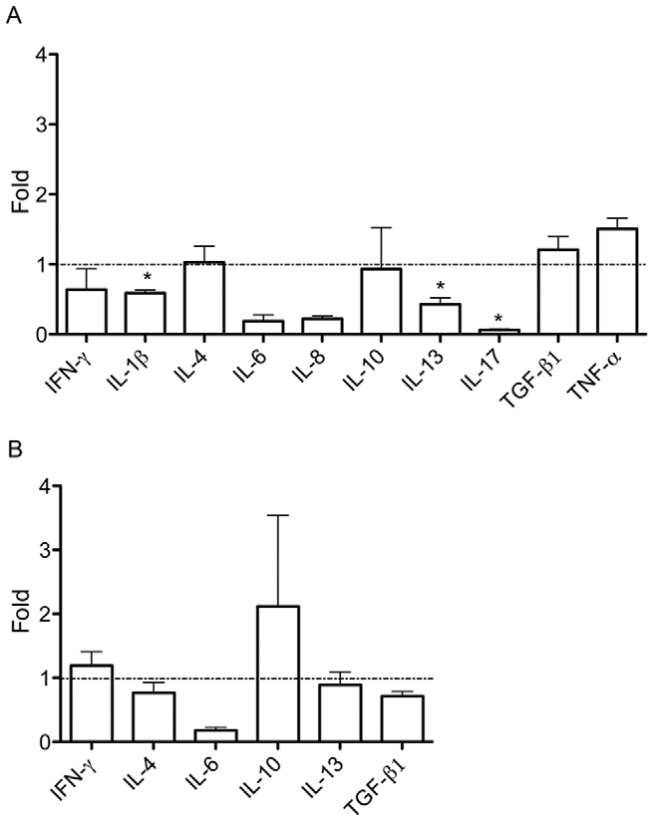
Cytokine expression profiles in jejunum and mesenterial lymph nodes of *Giardia duodenalis* infected calves. Transcription levels of the main cytokines were analyzed using quantitative PCR. Mean fold changes (n  = 4) when comparing infected animals to controls are presented with SEM as error bars. In jejunal tissue (panel A), no significant differences in transcription levels were found for INF-γ, IL-4, IL-6, IL-8, IL-10, TGF-β1 and TNF-α. The transcription of IL-13, IL-17, and IL-1β was significantly downregulated. In the draining mesenteric lymph nodes (panel B), there was no significant alternation of cytokine transcription. (* P<0.05).

Since the microarray data suggested a decrease in inflammation, immune response and immune cell migration, the intestinal immune responses in the animals were investigated in more detail by analyzing cytokine transcription levels combined with histological analyses, such as immune cell counts, quantification of apoptosis, and villus/crypt measurements. The results of the qRT-PCRs on a selected panel of cytokines are shown in Figure 3 as mean fold changes in infected animals compared to the control animals. In jejunal tissue, no significant differences in transcription levels were found for IFN-γ, IL-4, IL-6, IL-8, IL-10, TGF-β1, and TNF-α. The expression of IL-13, IL-17, and IL-1β was significantly downregulated with a fold change of 0.43, 0.06, and 0.59 respectively. There was no significant alteration of cytokine expression in the mesenteric draining lymph nodes of infected animals versus those of control animals. The expression levels of IL-10 seemed to be higher in infected than in control animals; although this difference was not significant.

Upon histological examination of the samples, the general morphology of the jejunal mucosa appeared to be normal without signs of inflammation. The results of the cell counts and villus/crypt measurements are summarized in Table 2 and 3. No significant differences were observed for T cells, B cells, mast cells, eosinophils, or the number of apoptotic cells between infected and control animals. Only the number of macrophages was significantly lower in infected animals (2.17 per 10^5^ µm) than in controls (3.17 per 10^5^ µm). A significant difference was also found for the villus/crypt ratio with an increased ratio in the infected animals (p<0.05).

**Table 2:** Histological cell counts in jejunal tissue off control animals and *Giardia duodenalis *infected calves.

	IEL^a^	T-cells^b^(CD3+)	B-cells^b^(CD20+)	Macrophages^b^(MAC387+)	Eosinophils^c^	Mast cells^c^
Control	23.2±4.1	240±37.5	2.6±1.3	3.2±0.6	549±295	23±19
Infected	18.7±3.1	217±23.1	4.7±2.7	2.2±0.2*	348±128	43±40

a intraepithelial lymphocytes per 100 enterocytes.

b number off cells per 10^5^ µm^2^.

c number off cells per 10HPF.

P≤0.05 versus control group, data are mean ± SEM.

**Table 3:** Villus and crypt measurements in jejunal tissue of control animals and *Giardia duodenalis* infected calves.

	Villus length (µm)		Crypt depth (µm)		Villus/crypt ratio	
	Mean ± sd	Range	Mean ± sd	Range	Mean ± sd	Range
Control	378±27	313–447	237±27	203–337	1.45±0.11	1.26–1.68
Infected	506±47	385–584	253±23	201–308	2.17±0.15*	1.91–2.59

* P≤0.05 versus control group.

## Discussion

Despite of *G. duodenalis* being an important and common parasite in humans and animals, our knowledge on host-parasite interactions in natural hosts is still limited. In this study, gene expression in the jejunum of uninfected and primary infected calves was analyzed using a whole genome microarray. This generated a list of functions and networks in IPA of which some can be related to suppression of inflammation and immunity. Further qRT-PCR analysis of a selected panel of cytokines revealed a trend of downregulated expression in infected animals compared to the negative controls,.In addition, no immune cell recruitment was detected in the intestine of the infected calves.

Microarray analyses identified the activation of PPARα and PPARγ as the possible regulators of the observed host responses. These nuclear receptors can be expressed in epithelial and endothelial cells as well as in immune cells such as T cells, B cells, dendritic cells, and macrophages [26]. The activation of PPARs can exert an anti-inflammatory effect by transrepressing the activity of several transcription factors, such as nuclear factor-κB (NF-κB) and activator protein 1 (AP1) [27]. Through this transrepression, the release of pro-inflammatory cytokines (IL-6, TNF-α, IL-12, IL-17, and IL-1β) and iNOS can be inhibited, as well as the recruitment of leukocytes [26], [28]. Whether the PPAR activation actually leads to the negative modulation of the inflammatory and immune response observed in the *Giardia* infected calves is still unclear. Further studies using PPAR agonists and antagonist are needed to provide direct evidence of the biological role of these receptors in the host response. Interestingly, the up-regulation of PPARγ was also observed in Caco-2 human intestinal epithelial cells after being incubated with *G. duodenalis* trophozoites for 18 hours [29].

How exactly PPAR activation occurs during the *Giardia* infection in these calves is unclear. The endogenous ligands of the PPARs are various fatty acids and metabolites of fatty acid metabolism that can be produced by inflammatory responses [27]. The ability to induce activation of PPARs was seen in other parasite infections such as *Plasmodium spp.*, *Toxoplasma gondii,* and protozoa of the *Leishmania* genus. *Plasmodium* spp. rupture red blood cells they infect. This leads to the formation of hemozoin, in its turn causing the release of the PPARγ ligand 15-hydroxyeicosatetraenoic acid (15-HETE). *T. gondii* induces the production of PPAR ligands by platelets, while visceral *Leishmania* infection induced PPARγ expression on residual macrophages, liver and spleen of mice [30]. A possible mechanism activating the PPARs during a *Giardia* infection might be the sphingolipids. These lipids have shown to be possible ligands for PPARs [31] and are also produced *de novo* by this parasite [32].

Besides the PPARs, other genes could be involved in the observed immune suppression, as function analysis by IPA indicated. An example is ADA, a gene that is upregulated in the infected animals and codes for adenosine deaminase. This enzyme is involved in the deamination of adenosine, a purine that can exert several effects on inflammation, both pro- and anti-inflammatory [33]. The end product of adenosine deamination is inosine, which in itself also has an anti-inflammatory effect [34].

Furthermore, no intestinal pathologies, such as villus shortening or increased levels of apoptosis were observed in the intestines of infected animals. The available information on pathophysiological changes induced by a *Giardia* infection in humans and animals is often contradictory. While some studies report histological changes in the villi and crypts and increased levels of intraepithelial lymphocytes in infected hosts [35], [36], others describe the lack of inflammation or other typical histological features in the majority of the patients investigated [37]. The reason for this discrepancy is still unclear, although some authors have suggested that assemblage-specific differences might exist. In a study by Chin et al. (2002) [38], the ability of *G. duodenalis* to induce apoptosis in enterocytes appeared to be strain dependent. In humans, patients infected with assemblage A are more likely to present clinical signs than those infected with assemblage B, although once established infections with assemblage B seem to result in more persistent diarrhea [39], [40]. Whether the general absence of pathologies or immune response in the current experiment was due to the infection with assemblage E needs to be further studied. Interestingly, Barigye et al. (2008) [41] previously described the absence of histological changes in the intestines of four out of five calves infected with assemblage E, supporting the observations made in this study. Alternatively, the presence or absence of inflammation, histological damage and/or clinical signs may depend on the infection dose. In this experiment, calves that were introduced into a contaminated housing excreted between 3950 and 15,000 CPG. While similar infection levels are often observed in field conditions, naturally infected calves can excrete up to 10^6^ CPG [42], [43].

Apart from the apparent immunosuppressive pathway induced by the *Giardia* infection, several genes involved in lipid metabolism were also impacted, such as genes coding for enzymes involved in lipid synthesis or lipolysis (PLA2G1B, CYP3A5, HPGD, ADA), transcription factors regulating the lipid metabolism (PPARα, PPARγ), and the ABC transporter ABCG8, responsible for efflux of cholesterol from enterocytes into the intestinal lumen or as high-density lipoproteins in plasma. Theoretically, the observed transcriptional changes would lead to a decreased uptake of lipids in the intestine and a higher efflux of cholesterol in the intestinal lumen. This host response could actually be beneficial for the parasite, since several studies have previously shown that some protozoa, including *Giardia,* are unable to synthesize the majority of their lipids and cholesterol *de novo* and rely on the host’s intestinal milieu for these products (reviewed by Das et al., 2002 [44]). Interestingly, it has been reported that Giardia-infected human patients often show intestinal fat malabsorption [45] and significantly lower levels of total serum cholesterol compared to healthy controls [46]. Whether this is the result of a similar intestinal response in humans as observed in cattle needs further research.

In conclusion, the outcome of the current study suggests that a primary *G. duodenalis* assemblage E infection in calves results in a decrease of inflammation, immune response and immune cell migration in the infected animals. This could explain the often chronic nature of a *G. duodenalis* infection in cattle and the lack of inflammation in the intestinal tissue. Whether *G. duodenalis* infections in other host species or *G. duodenalis* assemblage A infections in calves induce a similar intestinal response is still unclear and needs further research.

## Supporting Information

Table S1 **Genes used in the qRT-PCR assay, indicating GeneBank accession number and primer sequences.**
(DOCX)

Table S2 **Significantly regulated genes in bovine small intestine during a** ***Giardia duodenalis*** **infection.**
(DOCX)
