# Supplementary material for: Microarray Analysis of the Intestinal Host Response in Giardia duodenalis Assemblage E Infected Calves
Source: PLoS One. 2012 Jul 27;7(7):e40985. doi: 10.1371/journal.pone.0040985 (PMC3407150; doi:10.1371/journal.pone.0040985)
Supplement: Table S1 — Genes used in the qRT-PCR assay, indicating GeneBank accession number and primer sequences. (DOCX) [file pone.0040985.s001.docx]

**Table S1: Genes used in the qRT-PCR assay, indicating GeneBank accession number and primer sequences**

| **Gene symbol** | **Accesion number** | **Primer sequence** |
| --- | --- | --- |
| **Housekeeping genes** |  |  |
| *ACTB* | NM_173979.3 | F: GACATCCGCAAGGACCTCTA |
|  |  | R: ACATCTGCTGGAAGGTGGAC |
| *GAPDH* | NM_001034034.1 | F: ACCCAGAAGACTGTGGATGG |
|  |  | R: CAACAGACACGTTGGGAGTG |
| *HPRT1* | NM_001034035.1 | F: CACTGGGAAGACAATGCAGA |
|  |  | R: ACACTTCGAGGGGTCCTTTT |
| *RPLP0* | NM_001012682.1 | F: CTTCATTGTGGGAGCAGACA |
|  |  | R: GGCAACAGTTTCTCCAGAGC |
| *SDHA* | NM_174178.2 | F: ACATGCAGAAGTCGATGCAG |
|  |  | R: GGTCTCCACCAGGTCAGTGT |
| *UBE2D2* | NM_001046496.1 | F: AGCCAGTTCTCCAGGCATAA |
|  |  | R: TCTTCCCCATCCAAGAACAC |
| **Validation microarray** |  |  |
| *ABCG8* | NM_001024663 | F: GACCAGCATTGACAGACGAA |
|  |  | R: CACTCTGCTCCCTTGACTCC |
| *ADA* | NM_173887.2 | F: GACCTGGCTGGAGATGAGAC |
|  |  | R: CTCGGTCTTGAGTGTGTCCA |
| *FASN* | NM_001012669.1 | F:CTGAGTCGGAGAACCTGGAG |
|  |  | R: CATATTGTGTGCCTGCTTGG |
| *PPARA* | NM_001034036.1 | F:TCCCTCTTTGTGGCTGCTAT |
|  |  | R: TCGTCAGGATGGTTGTTCTG |
| *PPARG* | NM_181024.2 | F: GATCTTGACGGGAAAGACGA |
|  |  | R: ACTGACACCCCTGGAAGATG |
| *RASGRP2* | NM_001099946.1 | F: AGCCTGCTGATGGTGTCTTT |
|  |  | R: GTTGGCGAGGACTTAGAACG |
| *RHOD* | NM_001192338.2 | F: ATGTCACCAGTCCACACAGC |
|  |  | R: TCACCAGCATCTTGTCCTTG |
| **Cytokines** |  |  |
| *IFN-γ* | NM_174086.1 | F: TTCTTGAATGGCAGCTCTGA |
|  |  | R: TTCTCTTCGGCTTTCTGAGG |
| *IL-1β* | NM_174093.1 | F: AAGGCTCTCCACCTCCTCTC |
|  |  | R: TTTGGGGTCTACTTCCTCCA |
| *IL4* | NM_173921.2 | F: GCGGACTTGACAGGAATCTC |
|  |  | R: TCAGCGTACTTGTGCTCGTC |
| *IL-6* | NM_173923.2 | F: TCCTTGCTGCTTTCACACTC |
|  |  | R: CACCCCAGGCAGACTACTTC |
| *IL-8* | NM_173925.2 | F: GTTGCTCTCTTGGCAGCTTT |
|  |  | R: GGTGGAAAGGTGTGGAATGT |
| *IL-10* | NM_174088.1 | F: TGTTGACCCAGTCTCTGCTG |
|  |  | R: GGCATCACCTCTTCCAGGTA |
| *IL-13* | NM_174090.1 | F: GGTGGCCTCACCTCCCCAAG |
|  |  | R: ATGACACTGCAGTTGGAGATGCTG |
| *IL-17* | NM_001008412.1 | F: GGACTCTCCACCGCAATGAG |
|  |  | R: TGGCCTCCCAGATCACAGA |
| *TGF-β1* | NM_001166068.1 | F: CTGCTGTGTTCGTCAGCTCT |
|  |  | R: TCCAGGCTCCAGATGTAAGG |
| *TNF-α* | NM_173966.2 | F: GCCCTCTGGTTCAGACACTC |
|  |  | R: AGATGAGGTAAAGCCCGTCA |
