# Supplementary material for: Microarray Analysis of the Intestinal Host Response in Giardia duodenalis Assemblage E Infected Calves
Source: PLoS One. 2012 Jul 27;7(7):e40985. doi: 10.1371/journal.pone.0040985 (PMC3407150; doi:10.1371/journal.pone.0040985)
Supplement: Table S2 — Significantly regulated genes in bovine small intestine during a Giardia duodenalis infection. (DOCX) [file pone.0040985.s002.docx]

**Table S2**. **Significantly regulated genes in bovine small intestine during a *Giardia duodenalis* infection.**

| **Gene symbol** | **Gene name** | **Reference sequence ID** | **Fold Change** | **P-value** |
| --- | --- | --- | --- | --- |
| AGMO | Alkylglycerol monooxygenase | NM_001192973.1 | 17.70 | 0.0326 |
| ADA | Adenosine deaminase | NM_173887.2 | 9.21 | 0.0002 |
| LOC515676 |  | XM_593741.6 | 9.15 | 0.0113 |
| FGG | Fibrinogen gamma chain | NM_173911.2 | 8.87 | 0.0331 |
| SLC1A1 | Solute carrier family 1 glutamate | NM_174599.2 | 6.91 | 0.0276 |
|  | (neuronal/epithelial high affinity |  |  |  |
|  | transporter, system Xag), member 1 |  |  |  |
| LRAT | Lecithin retinol acyltransferase | NM_177503.2 | 6.84 | 0.0255 |
|  | (phosphatidylcholine--retinol O- |  |  |  |
|  | acyltransferase) |  |  |  |
| BOLA-DYB | Major histocompatibility complex, | NM_001012679.1 | 6.49 | 0.0380 |
|  | class II, DY beta |  |  |  |
| C19H17orf78 | Chromosome 19 open reading frame, | NM_001099158.1 | 5.59 | 0.0448 |
|  | human C17orf78 |  |  |  |
| SFTA2 | Surfactant associated 2 | NM_001114523.1 | 5.14 | 0.0010 |
| CYP2B | Cytochrome P450 subfamily 2B | NM_001075173.1 | 5.07 | 0.0218 |
| ACSM3 | Acyl-CoA synthetase medium-chain | XM_002697986 | 4.85 | 0.0376 |
|  | family member 3 |  |  |  |
| SLC23A1 | Solute carrier family 23 (nucleobase | XM_581784.5 | 4.15 | 0.0126 |
|  | transporters), member 1 |  |  |  |
| TKDP1 | Trophoblast Kunitz domain protein 1 | NM_205776.1 | 4.11 | 0.0478 |
| HGD | Homogentisate 1,2-dioxygenase | NM_001144852.1 | 3.70 | 0.0263 |
| DFNA5 | Deafness, autosomal dominant 5 | NM_001193112 | 3.54 | 0.0132 |
| AQP7 | Aquaporin 7 | NM_001076378.1 | 3.47 | 0.0143 |
| CYP3A5 | Cytochrome P450, family 3, | NM_001075888.1 | 3.40 | 0.0003 |
|  | subfamily A, polypeptide 5 |  |  |  |
| SULT2A1 | Sulfotransferase family, cytosolic, | NM_001046353.1 | 3.37 | 0.0182 |
|  | 2A, dehydroepiandrosterone |  |  |  |
|  | (DHEA)-preferring, member 1 |  |  |  |
| C9H6orf186 | Chromosome 9 open reading frame, | NM_001205452.1 | 3.31 | 0.0402 |
|  | human C6orf186 |  |  |  |
| CCL14 | Chemokine (C-C motif) ligand 14 | NM_001046585.1 | 3.26 | 0.0158 |
| ABCG8 | ATP-binding cassette, sub-family G | NM_001024663.1 | 3.17 | 0.0095 |
|  | (WHITE), member 8 |  |  |  |
| GPRIN3 | GPRIN family member 3 | XM_002688165 | 3.16 | 0.0242 |
| PMP22 | Peripheral myelin protein 22 | NM_001101156.1 | 3.11 | 0.0002 |
| RHOD | Ras homolog gene family, member D | NM_001192338.2 | 3.02 | 0.0015 |
| SLC34A2 | Solute carrier family 34 (sodium | NM_174661.2 | 2.90 | 0.0213 |
|  | phosphate), member 2 |  |  |  |
| SRGAP3 | SLIT-ROBO Rho GTPase activating | NM_001192966.1 | 2.89 | 0.0022 |
|  | protein 3 |  |  |  |
| NT5E | 5'-nucleotidase, ecto (CD73) | NM_174129.3 | 2.89 | 0.0117 |
| GMPR | Guanosine monophosphate reductase | NM_001075977.1 | 2.87 | 0.0022 |
| COBLL1 | COBL-like 1 | XM_002685374 | 2.84 | 0.0281 |
| PGA5 | Pepsinogen 5, group I (pepsinogen A) | NM_001001600.2 | 2.74 | 0.0057 |
| LOC505099 | Ankyrin repeat domain-containing | XM_003586746.1 | 2.70 | 0.0280 |
|  | protein 26-like |  |  |  |
| PC | Pyruvate carboxylase | NM_177946.3 | 2.63 | 0.0236 |
| MLPH | Melanophilin | NM_001081597.1 | 2.58 | 0.0251 |
| SLC16A13 | Solute carrier family 16, member 13 | NM_001076132.1 | 2.52 | 0.0269 |
|  | (monocarboxylic acid transporter 13) |  |  |  |
| PIK3C2G | Phosphoinositide-3-kinase, class 2, | NM_001206512.1 | 2.50 | 0.0146 |
|  | gamma polypeptide |  |  |  |
| HS3ST1 | Heparan sulfate (glucosamine) 3-O- | NM_001076122.1 | 2.48 | 0.0039 |
|  | sulfotransferase 1 |  |  |  |
| NEDD4 | Neural precursor cell expressed, | XM_584456.4 | 2.48 | 0.0015 |
|  | developmentally down-regulated 4 |  |  |  |
| HS3ST1 | Heparan sulfate (glucosamine) 3-O- | NM_001076122.1 | 2.48 | 0.0039 |
|  | sulfotransferase 1 |  |  |  |
| EFHD1 | EF-hand domain family, member D1 | NM_001075832.1 | 2.47 | 0.0371 |
| LOC514978 | Lipopolysaccharide-binding protein- | XM_592903.4 | 2.46 | 0.0119 |
|  | like |  |  |  |
| FIBIN | Fin bud initiation factor homolog | NM_001015541.1 | 2.43 | 0.0290 |
|  | (zebrafish) |  |  |  |
| PLA2G1B | Phospholipase A2, group IB | NM_174646.3 | 2.43 | 0.0353 |
|  | (pancreas) |  |  |  |
| HIST1H1D | Histone cluster 1, H1d | NM_001101066.1 | 2.42 | 0.0393 |
| ANKRD31 | Ankyrin repeat domain 31 | XM_593928.4 | 2.37 | 0.0145 |
| ANKRD40 | Ankyrin repeat domain 40 | NM_001075586.1 | 2.36 | 0.0328 |
| TFF2 | Trefoil factor 2 | NM_001083521.1 | 2.33 | 0.0331 |
| AKR1C4 | Allograft inflammatory factor 1-like | NM_181027.2 | 2.31 | 0.0127 |
| SOX13 | SRY (sex determining region Y)-box | XM_001255512.4 | 2.30 | 0.0307 |
|  | 13 |  |  |  |
| LOC528040 |  | XM_606449.6 | 2.26 | 0.0412 |
| TACSTD2 | Tumor-associated calcium signal | XM_589840.4 | 2.26 | 0.0409 |
|  | transducer 2 |  |  |  |
| PPARG-TSEN | Peroxisome proliferator-activated | NR_003097.1 | 2.23 | 0.0018 |
|  | receptor gamma |  |  |  |
| HIST1H1C | Histone cluster 1, H1c | NM_001083425.1 | 2.22 | 0.0085 |
| HPGD | Hydroxyprostaglandin dehydrogenase | NM_001034419.1 | 2.22 | 0.0395 |
|  | 15-(NAD) |  |  |  |
| SEPP1 | Selenoprotein P, plasma, 1 | NM_174459.3 | 2.22 | 0.0469 |
| FMO4 | Flavin containing monooxygenase 4 | NM_001192230 | 2.20 | 0.0118 |
| ENPP1 | Ectonucleotide pyrophosphatase | XM_867376.4 | 2.19 | 0.0019 |
|  | /phosphodiesterase 1 |  |  |  |
| ABHD4 | Abhydrolase domain containing 4 | NM_001034368.1 | 2.13 | 0.0049 |
| OTUD7B | OTU domain containing 7B | NM_001144093.1 | 2.13 | 0.0037 |
| LOC532189 |  | XR_027597.2 | 2.13 | 0.0227 |
| FGGY | FGGY carbohydrate kinase domain | NM_001192794 | 2.13 | 0.0209 |
|  | containing |  |  |  |
| RUNDC3B | RUN domain containing 3B | NM_001076874.1 | 2.13 | 0.0352 |
| FBXL2 | F-box and leucine-rich repeat protein | NM_001099153.1 | 2.12 | 0.0480 |
|  | 2 |  |  |  |
| ACE | Angiotensin I converting enzyme | NM_001206668.1 | 2.12 | 0.0456 |
| UGT2B15 | UDP glucuronosyltransferase 2 | XM_612336.5 | 2.12 | 0.0327 |
|  | family, polypeptide B17 |  |  |  |
| CHRNE | Cholinergic receptor, nicotinic, | NM_174272.2 | 2.10 | 0.0194 |
|  | epsilon |  |  |  |
| TNFSF15 | Tumor necrosis factor (ligand) | NM_001205782.1 | 2.10 | 0.0126 |
|  | superfamily, member 15 |  |  |  |
| RERGL | RERG/RAS-like | NM_001105472 | 2.09 | 0.0172 |
| CIB2 | Calcium and integrin binding family | XM_869807 | 2.09 | 0.0430 |
|  | member 2-like |  |  |  |
| STARD3NL | STARD3 N-terminal like | NM_001075427.1 | 2.08 | 0.0091 |
| SH3GL2 | SH3-domain GRB2-like 2 | NM_001098039.1 | 2.08 | 0.0305 |
| ALB | Albumin | NM_180992.2 | 2.07 | 0.0156 |
| LOC790332 | Butyrophilin, subfamily 1, member | XM_001256837.3 | 2.07 | 0.0422 |
|  | A1-like |  |  |  |
| EXPH5 | Exophilin 5 | XM_605435.4 | 2.06 | 0.0153 |
| OPLAH | 5-oxoprolinase (ATP-hydrolysing) | NM_001001173.1 | 2.05 | 0.0196 |
| SLC31A2 | Solute carrier family 31 (copper | NM_001034556.1 | 2.05 | 0.0209 |
|  | transporters), member 2 |  |  |  |
| PPARA | Peroxisome proliferator-activated | NM_001034036.1 | 2.03 | 0.0018 |
|  | receptor alpha |  |  |  |
| DENND1C | DENN/MADD domain containing 1C | NM_001193164 | -2.00 | 0.0347 |
| SLC12A8 | Solute carrier family 12 | XM_599718.5 | -2.00 | 0.0107 |
|  | (potassium/chloride transporters), |  |  |  |
|  | member 8 |  |  |  |
| PDE6A | Phosphodiesterase 6A, cGMP- | NM_001001526.2 | -2.02 | 0.0020 |
|  | specific, rod, alpha |  |  |  |
| FASN | Fatty acid synthase | NM_001012669.1 | -2.04 | 0.0009 |
| CDC42EP2 | CDC42 effector protein (Rho GTPase | NM_001075341.1 | -2.04 | 0.0196 |
|  | binding) 2 |  |  |  |
| GNG4 | Guanine nucleotide binding protein | NM_001103324.1 | -2.04 | 0.0148 |
|  | (G protein), gamma 4 |  |  |  |
| HSPH1 | Heat shock 105kDa/110kDa protein 1 | NM_001075302.1 | -2.04 | 0.0311 |
| ITIH4 | Inter-alpha-trypsin inhibitor heavy | NM_001015590.2 | -2.04 | 0.0203 |
|  | chain family, member 4 |  |  |  |
| ANO8 | Anoctamin 8 | XM_002688544.2 | -2.04 | 0.0051 |
| ARHGAP4 | Rho GTPase activating protein 4 | NM_001102540.1 | -2.08 | 0.0399 |
| IL12RB1 | Interleukin 12 receptor, beta 1 | XM_602621.4 | -2.08 | 0.0288 |
| MAP4K1 | Mitogen-activated protein kinase | NM_001075825.2 | -2.08 | 0.0216 |
|  | kinase kinase kinase 1 |  |  |  |
| MYO1G | Myosin IG | XM_594668.4 | -2.09 | 0.0001 |
| SPINK1 | Serine peptidase inhibitor, Kazal type | NM_001025348.1 | -2.10 | 0.0092 |
|  | 1 |  |  |  |
| CD1B | CD1b molecule | NM_001046021.1 | -2.13 | 0.0125 |
| SLC9A3R2 | Solute carrier family 9 | NM_001077065.1 | -2.13 | 0.0148 |
|  | (sodium/hydrogen exchanger), |  |  |  |
|  | member 3 regulator 2 |  |  |  |
| ADAM19 | ADAM metallopeptidase domain 19 | NM_001075475 | -2.17 | 0.0435 |
| COL21A1 | Collagen, type XXI, alpha 1 | NM_001102156.1 | -2.17 | 0.0366 |
| C2H2orf72 | Chromosome 2 open reading frame, | XM_001254835.4 | -2.17 | 0.0246 |
|  | human C2orf72 |  |  |  |
| PIEZO1 | Piezo-type mechanosensitive ion | XM_002694859.2 | -2.17 | 0.0102 |
|  | channel component 1 |  |  |  |
| LRP8 | Low density lipoprotein receptor- | NM_001097565.1 | -2.17 | 0.0183 |
|  | related protein 8, apolipoprotein E |  |  |  |
|  | receptor |  |  |  |
| RASGRP2 | RAS guanyl releasing protein 2 | NM_001099946.1 | -2.21 | 0.0047 |
|  | (calcium and DAG-regulated) |  |  |  |
| AIF1L | Allograft inflammatory factor 1-like | NM_001078079.1 | -2.22 | 0.0390 |
| MAP3K6 | Mitogen-activated protein kinase | NM_001193236.1 | -2.23 | 0.0064 |
|  | kinase kinase 6 |  |  |  |
| FSTL3 | Follistatin-like 3 (secreted | NM_001075710.1 | -2.27 | 0.0322 |
|  | glycoprotein) |  |  |  |
| SOX1 | SRY (sex determining region Y)-box | XM_870462.1 | -2.30 | 0.0087 |
|  | 1 |  |  |  |
| BCAT1 | Branched chain amino-acid | NM_001083644.1 | -2.33 | 0.0457 |
|  | transaminase 1, cytosolic |  |  |  |
| ITGA4 | Integrin, alpha 4 (antigen CD49D, | NM_174748.1 | -2.33 | 0.0500 |
|  | alpha 4 subunit of VLA-4 receptor) |  |  |  |
| ERP27 | Endoplasmic reticulum protein 27 | NM_001038041.1 | -2,38 | 0.0374 |
| DDX51 | DEAD (Asp-Glu-Ala-Asp) box | XM_002694436 | -2.38 | 0.0280 |
|  | polypeptide 51 |  |  |  |
| OSBPL7 | Oxysterol binding protein-like 7 | NM_001205647.1 | -2.38 | 0.0426 |
| SH2D3C | SH2 domain containing 3C | NM_001098039.1 | -2.38 | 0.0205 |
| SLC17A9 | Solute carrier family 17, member 9 | NM_001100378.1 | -2.40 | 0.0342 |
| BLK | B lymphoid tyrosine kinase | NM_001075968.1 | -2.44 | 0.0215 |
| CLSTN3 | Calsyntenin 3 | NM_001075425.2 | -2.44 | 0.0379 |
| LOC100137901 |  | XR_083492.2 | -2.46 | 0.0017 |
| NTRK3 | Neurotrophic tyrosine kinase, | XM_585006.4 | -2.50 | 0.0245 |
|  | receptor, type 3 |  |  |  |
| F13A1 | Coagulation factor XIII, A1 | NM_001167894 | -2.50 | 0.0083 |
|  | polypeptide |  |  |  |
| CCDC69 | Coiled-coil domain containing 69 | NM_001206624 | -2.56 | 0.0112 |
| CPXM1 | Carboxypeptidase X (M14 family), | NM_001015642.2 | -2.63 | 0.0056 |
|  | member 1 |  |  |  |
| MADCAM1 | Mucosal vascular addressin cell | NM_001037821.1 | -2.63 | 0.0272 |
|  | adhesion molecule 1 |  |  |  |
| MCAM | Melanoma cell adhesion molecule | NM_001110062.1 | -2.63 | 0.0376 |
| NOL6 | Nucleolar protein family 6 (RNA- | NM_001099185.1 | -2.63 | 0.0497 |
|  | associated) |  |  |  |
| SPINK1 | Serine peptidase inhibitor, Kazal type | NM_001025348.1 | -2.83 | 0.0092 |
|  | 1 |  |  |  |
| NOS2 | Nitric oxide synthase 2, inducible | NM_001076799.1 | -2.94 | 0.0400 |
| POLR1C | Polymerase (RNA) I polypeptide C, | NM_001038124.1 | -2.94 | 0.0258 |
|  | 30kDa |  |  |  |
| SLC17A9 | Solute carrier family 17, member 9 | NM_001100378.1 | -2.94 | 0.0342 |
| IL21R | Interleukin 21 receptor | NM_001193179 | -3.13 | 0.0277 |
| CD79B | CD79b molecule, immunoglobulin- | XM_002696068 | -3.13 | 0.0210 |
|  | associated beta |  |  |  |
| CNTNAP1 | Contactin associated protein 1 | XM_001788969.1 | -3.13 | 0.0007 |
| DEF5B | Defensin, beta 5 | NM_001130761.1 | -3.70 | 0.0450 |
| CXCR5 | Chemokine (C-X-C motif) receptor 5 | NM_001011675.1 | -4.00 | 0.0394 |
| DEFB4A | Defensin, beta 4A | NM_174775.1 | -4.76 | 0.0305 |
| POU2AF1 | POU class 2 associating factor 1 | NM_001075915.1 | -5.00 | 0.0167 |
| MS4A1 | Membrane-spanning 4-domains, | NM_001077854.1 | -7.69 | 0.0484 |
|  | subfamily A, member 1 |  |  |  |
| CXCL13 | Chemokine (C-X-C motif) ligand 13 | NM_001015576.2 | -20.00 | 0.0303 |

Numbers represent fold with control= 1.0. The values below 1.0 indicate a downregulation.
